# Development and Validation of the COVID-19 Knowledges and Behavior Questionnaire in a French Population (CoVQuest-CC)

**DOI:** 10.3390/ijerph19052569

**Published:** 2022-02-23

**Authors:** Elise Verot, Paul Bonjean, Robin Chaux, Julie Gagnaire, Amandine Gagneux-Brunon, Bruno Pozzetto, Philippe Berthelot, Elisabeth Botelho-Nevers, Franck Chauvin

**Affiliations:** 1 Laboratoire Parcours Santé Systémique, Université Claude Bernard Lyon 1, Université de Lyon, P2S UR 4129, F-69008 Lyon, France; franck.chauvin@univ-st-etienne.fr; 2 Equipe PREDUCAN, CIC Inserm 1408 Saint-Etienne, F-42055 Saint-Etienne, France; 3 Chaire Hygée, Institut PRESAGE, Université Jean Monnet, Université de Lyon, F-42023 Saint-Etienne, France; 4 Laboratoire Parcours Santé Systémique, Université Jean Monnet, Université de Lyon, P2S UR 4129, F-42270 Saint-Etienne, France; 5 Unité de Recherche Clinique, Innovation, Pharmacologie, Hôpital Nord, Centre Hospitalier Universitaire de Saint-Étienne, F-42055 Saint-Etienne, France; robinvchaux@gmail.com; 6 Team GIMAP, CIRI—Centre International de Recherche en Infectiologie, Université Jean Monnet, Université de Lyon, Université Claude Bernard Lyon 1, Inserm, U1111, CNRS, UMR530, F-42023 Saint-Etienne, France; julie.gagnaire@chu-st-etienne.fr (J.G.); amandine.gagneux-brunon@chu-st-etienne.fr (A.G.-B.); bruno.pozzetto@chu-st-etienne.fr (B.P.); philippe.berthelot@chu-st-etienne.fr (P.B.); elisabeth.botelho-nevers@chu-st-etienne.fr (E.B.-N.); 7 Service d’Infectiologie, CIC Inserm 1408, Centre Hospitalier Universitaire de Saint-Étienne, F-42055 Saint-Etienne, France; 8 Chaire PreVacCI, Institut PRESAGE, Université Jean Monnet, Université de Lyon, F-42023 Saint-Etienne, France; 9 Laboratoire des Agents Infectieux, Centre Hospitalier Universitaire de Saint-Étienne, F-42055 Saint-Etienne, France

**Keywords:** COVID-19, SARS-CoV-2, health literacy, behaviors, questionnaire, mass screening

## Abstract

Background: The SARS-CoV-2 pandemic has led most countries to take restrictive measures affecting social activities and individual freedoms to limit viral transmission. It was shown that practical, motivational and social barriers impact on adherence to the isolation and social distancing measures advocated by the health authorities. The purpose of this study was to develop and validate a COVID-19 Knowledges and Behavior Questionnaire adapted to a teenager and adult French population. Methods: CoVQuest-CC was developed by a multidisciplinary team made of infectious diseases physicians, medical virologist, specialists of infectious control, experts of the questionnaires methodology, experts in public health and prevention, and statisticians. CoVQuest-CC was responded to by a big cohort from the general population during their participation in a massive SARS-CoV-2 screening campaign in 2021 in Saint-Etienne, France. Results: The confirmatory factorial analysis yielded good results (CFI = 0.94, TLI = 0.94, RMSEA = 0.04), and confirmed the five-dimensional structure of the questionnaire. Each dimension had a satisfying internal consistency, with Cronbach alphas of 0.83, 0.71, 0.65, 0.72 and 0.83 for transmission knowledge, barrier gesture respect, tests acceptability, home isolation possibility and test practicability, respectively. Conclusions: According to our knowledge, CoVQuest-CC is the first validated, consistent and reliable self-administrated French-specific questionnaire investigating the general population’s knowledge and attitudes towards COVID-19. It shows acceptable psychometric properties and can be use by Public Health teams or caregivers for public health and research purposes. Trial Registration: The study protocol was approved by the IRB ILE-DE-FRANCE 1 (No. IRB: I ORG0009918). All participants were given written and verbal information about the study and gave informed consent to participate. ClinicalTrials.gov identifier (NCT number): NCT04859023.

## 1. Introduction

The recent emergence of SARS-CoV-2 at the end of 2019 has led to a pandemic, resulting in exceptional health and restriction measures on all continents, including the lockdown of the entire populations of countries. The numbers of deaths, the massive number of hospitalizations and the resulting saturation of health care systems have led most countries to take restrictive measures affecting social activities and individual freedoms. These very restrictive measures aim to limit interactions between individuals and thus limit viral transmission [1]. Following the example of other countries, France had to implement various sanitary measures, binding for the population, within the framework of a specific public health policy to reduce the circulation of the virus. Thus, since March 2020, France has experienced three episodes of lockdown and various episodes of curfews, the last of which has just ended in June 2021. The government’s “Test–Alert–Protect” strategy aims to break the chains of transmission. Digital tools are available online to support the population in this process.

However, despite all the strategies put in place in conjunction with public awareness campaigns, there is still a significant number of individuals who do not strictly follow the recommendations put in place to combat the spread of the virus [2] Research on health behavior change has shown that more than a third of the European population has difficulties in finding, understanding, evaluating and using the information needed to manage their health [3]. Various studies show that low levels of health literacy lead to lower awareness of prevention, higher prevalence of health risk factors and problems in understanding medical instructions [4,5,6].

In the context of information campaigns on SARS-CoV-2, including health measures to protect against the virus, it has recently been suggested that people pay particular attention to the health literacy of the population receiving the information [2,7] It is important to remain vigilant about the need to adapt communication for people with lower levels of health literacy [2,7]. Moreover, the health crisis has shown that practical, motivational and social barriers impact on adherence to the isolation and social distancing measures advocated by the health authorities [4]. Spring rightly points out that, without a good health culture, people are not able to effectively distinguish between fact and fiction and may allow unreliable information to influence their behavior. This can be detrimental not only to the individual, but to society as a whole [8].

In the control of epidemics, beyond mass non-pharmaceutical interventions by cities or governments, each citizen also has a role, and can be an actor. Indeed, a good understanding and knowledge of the mode of transmission of SARS-CoV-2, adherence to stricter measures and rules, and the realistic possibility for individuals to isolate themselves are also part of pandemic control. In addition, after mass testing is initiated, or even after a negative result is obtained, efforts to continue to protect oneself and others must continue. As West et al. highlighted in May 2020, there is an urgent need for effective interventions to increase the general population’s adherence to the proper implementation of health measures to protect themselves individually and collectively [9]. Matterne et al. have highlighted the importance of developing and validating instruments that will measure health literacy related to COVID-19 [10]. Since the beginning of the pandemic, numerous studies around the world have investigated the knowledge, attitudes and habits of individuals (health professionals or specific strata of the general population), with respect to COVID-19, including the possible link with health literacy [11,12,13,14,15,16]. Most of these studies conducted surveys or used instruments validated in their language and culture.

As part of the French health policy in 2021, the city of Saint-Etienne (located in the Rhône Alpes Auvergne region, France) launched a vast screening operation to detect SARS-CoV-2 in asymptomatic volunteers in the general population [17]. Because a citywide assessment of knowledge, behavior, and isolation options for SARS-CoV-2 infection was not undertaken, we took the opportunity to add a research protocol to this mass screening operation. Thus, our research team wished to carry out a study on the knowledge and behavior of individuals regarding the transmission of the virus, the health measures in force and the motivations to go for screening. to the best of our knowledge, no questionnaire in French was designed or validated to establish such measures. Therefore, we decided to develop the COVID-19 Knowledges and Behavior Questionnaire (CoVQuest-CC) to assess the level of knowledge of the population on the transmission of the virus, the barrier measures, the behaviors regarding adherence to the barrier measures and the modalities of isolation in the case of a positive test. Therefore, the purpose of this study was to develop and validate a COVID-19 and Behavior Questionnaire adapted to a teenager and adult French population.

## 2. Materials and Methods

The development of the questionnaire and validation study took place in two phases.

The first phase consisted of developing the CoVQuest-CC questionnaire in French. The second phase was related to the validation of the questionnaire.

### 2.1. Questionnaire Development

The questionnaire was necessarily developed and tested before the mass population screening campaign. Based on various studies that have developed and validated questionnaires related to health knowledge, perceptions and behaviors and/or Knowledge, Attitudes and Practices (KAP), we proceeded as follows to develop this questionnaire [18,19,20,21,22].

We brought together ten experts: three infectious diseases physicians and one medical virologist, two specialists of infectious control, one expert of questionnaires methodology, two experts in public health and prevention and one statistician.

The first step was to generate the items to be included in the questionnaire. This was based on a literature review and the results of a brainstorming session with the group of experts.

Then, the expert group proceeded, based on a consensus, with the work of the classification—grouping and categorization—of the items to reduce them. Finally, the expert group was able to generate the questionnaire, again in a collegial manner based on consensus.

Four concept areas were isolated:

(1) Knowledge of the modes of transmission of SARS-CoV-2;

(2) Individual behavior regarding the respect of barrier measures;

(3) Individual capacity to implement the recommendations of the French health authorities in the event of a positive result;

(4) Individual acceptability of the various screening tests validated by the French health authorities.

Given that the purpose of the questionnaire is to measure an individual’s attitude and knowledge, the choice was made to use a Likert scale as the response method. This response mode consists of one or more statements (statements or items) for which the respondent expresses his/her degree of agreement or disagreement [23].

For the concept areas 1, 2 and 3, all response modalities are offered in the form of a Likert scale. Only concept area number 4 contains a mix of response modalities according to the typology of the questions asked. Indeed, out of 15 questions, 5 of the questions are related to the individual’s attitude and therefore retain the Likert scale of the 3 previous concept areas. Concerning the 10 additional questions relating to a choice of screening test format as well as to a representation of the pain inherent in the test format, 6 include response modalities in the form of a numerical scale (Visual Analogue Scale type) and 4 are presented in a categorical form, 3 of which require some free expression.

The precise content of the CoVQuest-CC questionnaire is presented in Table A1 (Appendix A), as are the acronyms used in this article to refer to each question.

Find below an overview of the main content of the questionnaire (Table 1).

The questionnaire was then pretested with 18 participants: 10 health professionals and 8 individuals from the general population, outside the medical or health world, including 3 minors (10, 13 and 17 years old). After each pretest, the tester was asked to discuss and interpret each question in the questionnaire. After this step was made, it was possible to compare the variability between answers, the understanding of the questions and their wording, and any ambiguity. All feedback from testers was considered to enable us to produce a revised, final version of the CoVQuest-CC.

### 2.2. Validation of the Questionnaire

A total of 8045 persons responded to the questionnaire. They were recruited during the mass screening campaign proposed to the population of Saint-Etienne (170,000 inhabitants) from 13 to 19 January and 22 to 28 February 2021. Thus, 3338 were recruited in the first wave of mass screening and 4707 during the second wave of mass screening.

Screenings were offered for free at 12 ephemeral sites, and in parallel, mobile teams were deployed to target populations (adolescents, students, people living in low-income neighborhoods, businesses, etc.). People wishing to participate in the study had to be over 10 years-old and able to read and understand the French language. For minors, parental permission was required.

No financial compensation was given to participants. This study was conducted in accordance with the guidelines set out in the Declaration of Helsinki. The research in which this validation took place was approved by the Ethics Committee of IRB ILE-DE-FRANCE 1 (*n*° IRB: I ORG0009918) and all participants gave their written consent.

#### Statistical Analysis for Psychometric Validation

First of all, Likert-type categorical questions were converted into numerical variables (details of this conversion are available in Appendix A—Table A1).

Then a descriptive analysis was performed using numbers and proportions for categorical variables and using mean, median, standard deviation, range and interquartile range for numerical variables. The next steps of psychometric validation were carried out only on the February responses, since the acceptability section was available only in February. Moreover, all questions presented in Table A1 were integrated in this validation, except for Accept_9, Accept_11 and Accept_13, as these questions were necessarily dependent to other question responses and were rather inserted for a qualitative approach. Subjects with missing data on interest variables were removed from the following analysis.

The data were randomly split in a 1:1 ratio. The first set was used as a training set (*n* = 2353) on which the exploratory factorial analysis (EFA) was conducted. The second set was used as a validation set (*n* = 2354) on which the confirmatory factorial analysis (CFA) was conducted.

The first step of the EFA was intended to assess the factorability of the survey. to this end, the Kaiser–Meyer–Olkin (KMO) Measure of Sampling Adequacy was computed to review the strength of the partial correlation between the variables. The suggested cut-off for determining the factorability of the sample data is a KMO ≥ 0.6, and the optimal cut-off is a KMO ≥ 0.8. Additionally, the Bartlett’s test of Sphericity was computed to test the overall correlations between the survey’s questions. A significant statistical test (*p* < 0.05) shows that the correlation matrix is not an identity matrix and is fit for a factorial analysis.

Potential redundancy between items was evaluated using a primary component analysis (PCA) and a Pearson correlation matrix. Redundancy was suspected between two questions when they were spatially close in PCA and had simultaneously a correlation coefficient over 0.6. In this case the two items were analyzed again by experts to assess if the items were only correlated, or truly redundant (because they display the same information). In the latter case we selected and retained only one of the redundant items.

To explore the factorial structure of the survey, we followed the method described by Terwee et al. [24]. A graphical estimate of the number of dimensions was first performed using a scree plot displaying eigenvalues [25]. A parallel analysis with 100 simulations was added on this plot and the number of dimensions was considered equal to the number of eigenvalues positioned significantly higher than the simulation line [26].

Then a factorial analysis with varimax rotation using the previously determined number of dimensions was performed. An item was considered associated with a factor if its loading was superior to 0.3 [27]. In case of items associated with several factors, the item was considered to belong to the factor with the highest loading value. Items not associated with any factor were not included in the CFA.

The CFA was conducted by structural equation modeling, with the marker index method [28] using the structure of the factorial model developed in the EFA. The item loadings and factorial structure were analyzed again, this time with the validation dataset. Items were considered poor in case of cross-loadings (loadings > 0.4 across two or more factors) and/or loadings < 0.4 [27]. Items considered poor were not immediately excluded; the loading value could be overruled and the item kept in the factor after expert’s assessment of the interest of the item.

Goodness of fit of the factorial models was assessed by computing the Comparative Fit Index (CFI), with a target value ≥ 0.90 deemed as an indicator of good fit [29]; the Tucker–Lewis Index (TLI), with a target value > 0.95 [30] as an indicator of good fit; and the root mean square error of approximation (RMSEA) and its 95% CI, with a target value ≤ 0.05 [30] as an indicator of good fit. The final factorial model was selected by minimizing the sample size adjusted Bayesian Information Criteria (BIC).

To assess internal consistency, Cronbach alpha coefficients and their 95% confidence intervals were calculated for each factor identified by the final CFA model [31]. A Cronbach alpha ≥ 0.70 for a factor was considered acceptable, and ≥0.90 excellent [32]. Finally, for dimensions with satisfying internal consistency results a global score was calculated by summing the scores of their component items. This global score was used to search for potential floor and ceiling effects.

Analyses were performed using R, a language and environment for statistical computing (R Core Team (2021). R Foundation for Statistical Computing, Vienna, Austria. URL https://www.R-project.org/, accessed on 27 July 2021. version 4.0.3 (with the packages “psy”, version 1.1, “lavaan” version 0.6–10, and “psych” version 2.1.9)).

## 3. Results

### 3.1. Participants Characteristics

The table below presents the main socio-demographic characteristics of the participants (see Table 2).

### 3.2. Face Validity, Content Validity and PreTest Phase

Face and content validity were considered satisfying by the multidisciplinary panel of experts. The pretest phase showed no argument for misunderstanding or ambiguity, and showed a sufficient variability between respondents’ answers. Besides this, the three questions about test acceptability, including free-form text responses (Accept_9, Accept_11, Accept_13), showed no incoherent responses in distributed questionnaires.

Additionally, a strong floor effect was found on item Accept_3, corresponding to pain assessment for saliva collection, which is not relevant as the test itself is by nature painless.

Four items had more than 14% non-response: Isol_5, Barriere_7, Accept_1 and Accept_2. These questions are determined by certain conditions, such as having children, being currently unemployed, and having had a nasopharyngeal swab. Thus, a “not applicable” answer modality to those items must be added.

### 3.3. Redundancy Assessment

**Figure A1:**
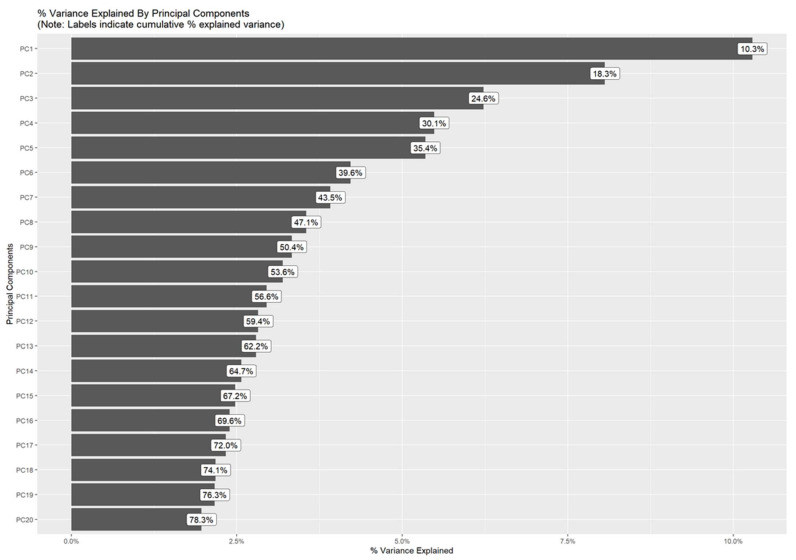
PCA: Variance percentages associated with each principal component.

**Figure A2:**
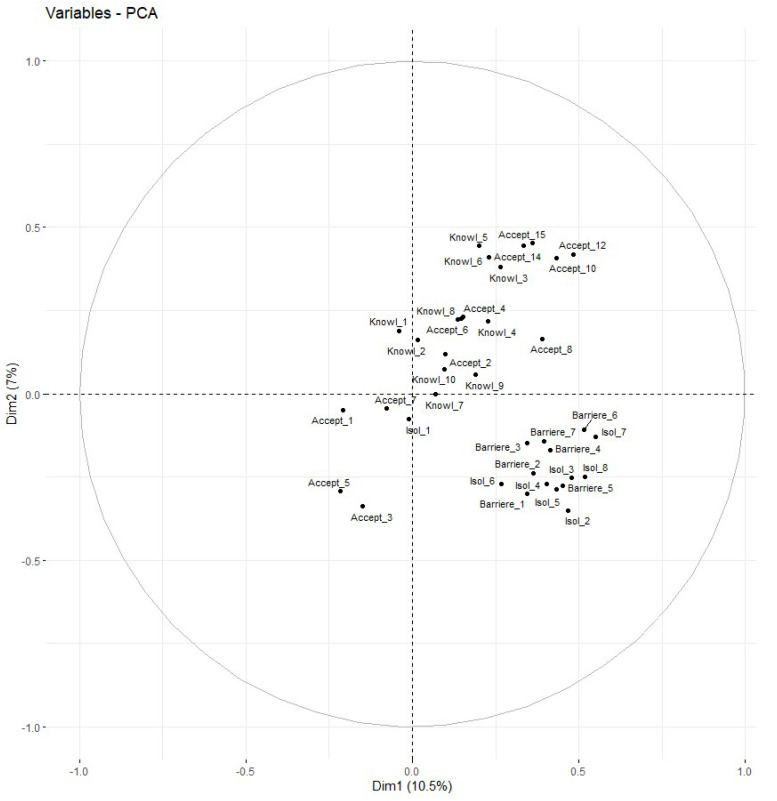
Item positions in PCA.

**Figure A3:**
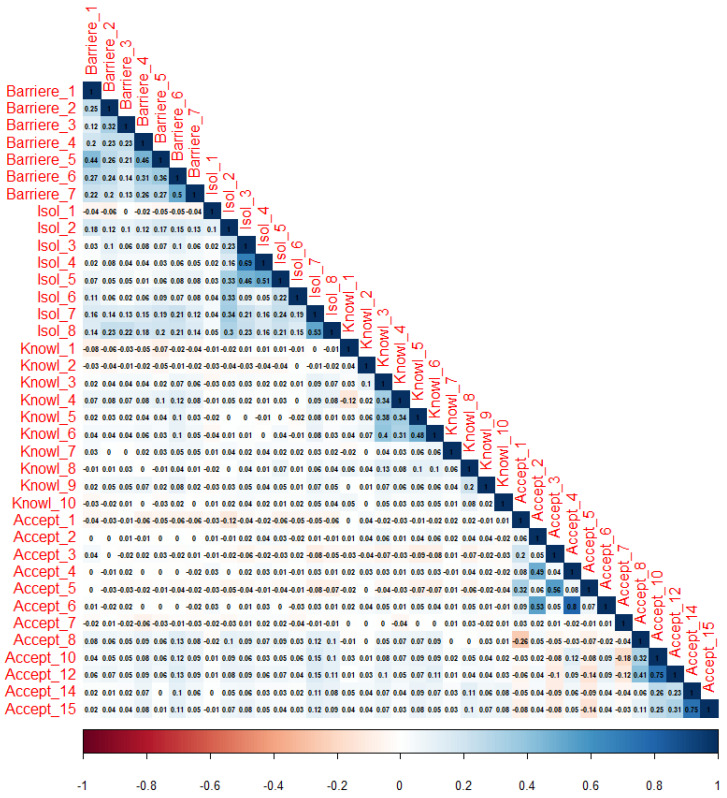
Correlation matrix between items.

Redundancy was assessed for the 4707 responses collected in February. A two-axis PCA was chosen based on the barplot representing variance proportions explained by principal components (Figure A1), the first two axes representing 18.3% of total variance. According to the PCA (Figure A2) and the correlation matrix results (Figure A3), redundancy was identified between Isol_3 and Isol_4, Knowl_5 and Knowl_6, Accept_4 and Accept_6, Accept_10 and Accept_12, and Accept_14 and Accept_15. Following consultation with the experts, Accept_14 was the only question considered truly redundant. Thus, Accept_14 was not included in the following analysis.

### 3.4. Exploratory Factorial Analysis

On the training dataset, the KMO Measure of Sampling Adequacy was equal to 0.73, and the significance of the Bartlett’s test of Sphericity was <0.001. Both indicators suggest that the data might be well-suited for a factor analysis.

**Figure A4:**
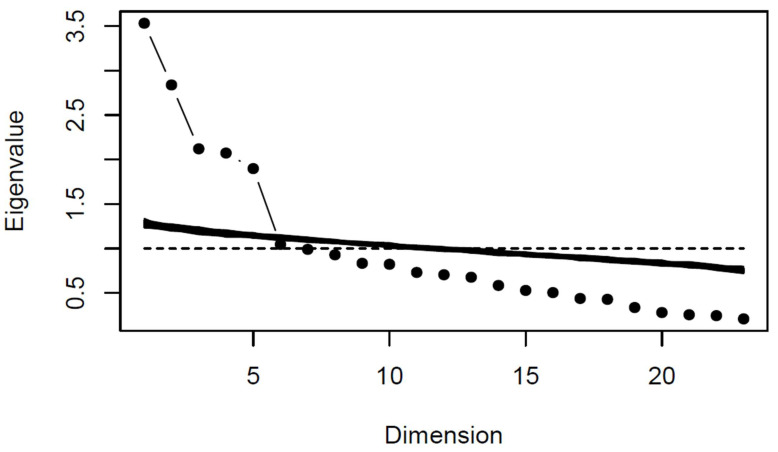
Eigenvalues diagram with 100 simulations.

The scree plot was in favor of an item repartition into five distinct dimensions (Figure A4). The exploratory factorial analysis showed that 8 of the 35 items explored were not associated with any of the dimensions at the loading cut-off of 0.3. After removing those items, the five-factor structure accounted for 40.0% of the training data variance.

### 3.5. Confirmatory Factorial Analysis

After EFA, iterations of CFA and experts feedback, the best model was a first-order, five-factor structural model. The five identified dimensions were (Table 3):

1—The first dimension included six items—Knowl_2, Knowl_3, Knowl_4, Knowl_5, Knowl_6, Knowl_8—and was therefore assumed to represent “SARS-CoV-2 transmission knowledge”;

2—The second dimension included six items—Barriere_1, Barriere_2, Barriere_4, Barriere_5, Barriere_6, Barriere_7—and was therefore assumed to represent “Barrier gestures respect”;

3—The third dimension included four items—Accept_8, Accept_10, Accept_12, Accept_15—and was therefore assumed to represent “Tests screening acceptability”;

4—The fourth dimension included four items—Isol_2, Isol_3, Isol_4, Isol_5—and was therefore assumed to represent “Home isolation possibility”;

5—The fifth dimension included three items—Accept_2, Accept_4, Accept_6—and was therefore assumed to represent “Test practicability”.

For this last model, the goodness-of-fit indices are satisfactory, with a CFI of 0.944, a TLI of 0.935, and an RMSEA of 0.043 with a 95% CI of 0.039–0.047.

Internal consistency was then assessed for each factor (Table 3). The first factor had a Cronbach α of 0.83, with a 95% confidence interval of 0.81–0.84. The second factor had a Cronbach α of 0.71, with a 95% confidence interval of 0.69–0.73. The third factor had a Cronbach α of 0.65, with a confidence interval of 0.63–0.69. The fourth factor had a Cronbach α of 0.72, with a 95% confidence interval of 0.69–0.74. The fifth factor had a Cronbach α of 0.83, with a confidence interval of 0.81–0.84. All factors had a Cronbach α > 0.7, except the third, with a Cronbach α = 0.65. However, this set of results suggests a satisfactory internal consistency, and the possibility of scoring items within each factor.

### 3.6. Global Score Calculations and Search for Flooring and Ceiling Effects

The global score of the “SARS-CoV-2 transmission knowledge” dimension had a median of 5, an interquartile range (IQR) of 4–5.5, a minimum of 0 and a maximum of 6. After numeric and graphic assessment, no floor or ceiling effects were found.

The global score of the “Barrier gestures respect” dimension had a median of 18, an IQR of 15–21, a minimum of 1 and a maximum of 24. After numeric and graphic assessment, no floor or ceiling effects were found.

The global score of “Tests screening acceptability” had a median of 11, an IQR of 9–12, a minimum of 0 and a maximum of 12. The numeric and graphic assessment suggests a ceiling effect, which, along with a Cronbach α of 0.65, suggests that it is the weakest factor in the questionnaire.

The global score of the “Home isolation possibility” dimension had a median of 5, an IQR of 3–7, a minimum of 0 and a maximum of 16. After numeric and graphic assessment, no floor or ceiling effects were found.

Finally, the global score of the “Test practicability” dimension had a median of 20, an IQR of 10–25, a minimum of 0 and a maximum of 30. After numeric and graphic assessment, no floor or ceiling effects were found.

## 4. Discussion

In this study, we report on the development and validation of the first French self-administered questionnaire on the knowledge and behavior of the general population regarding SARS-CoV-2 (modes of transmission and prevention measures, among others) in a large predominantly urban population. The CoVQuest-CC is a practical scoring questionnaire, useful for Public Health practice and research. This questionnaire allows one to quickly screen the knowledge profile of individuals regarding the modes of transmission, and the individual and collective protection measures regarding SARS-CoV-2. It also allows one to quickly screen individual behaviors regarding the adoption of sanitary measures in force.

The development and validation process suggest the satisfying validity of this questionnaire. First, the questionnaire was elaborated through discussion between a multidisciplinary panel of experts until a consensus was obtained about face validity and content validity. The pretest phase showed no sign of misunderstanding, even if more qualitative data would be necessary to make sure that question comprehension was sufficient. As this questionnaire also aimed to measure a global level of knowledge and behaviors related to SARS-CoV-2 and a global acceptability of its diagnostic tests, an exploratory and confirmatory factorial analysis were performed on training and test datasets to select the most pertinent questions and assess the factorial structure of the questionnaire.

However, the validation process presents several deficiencies. Since the study was principally designed to offer SARS-CoV-2 screening tests quickly and in convenient conditions, it was not considered to ask participants to respond again to the CoVQuest-CC questionnaire, and therefore it was not possible to assess its reproducibility. For the same reason, the choice was made to ask only a few questions about respondent’s socioeconomic conditions, thus it was not possible to assess discriminant validity regarding the various hypotheses on population knowledge and behaviors linked with housing area or socio-economic level, among others we found in the literature [13,33,34,35]. to our knowledge, there are no other validated instruments to measure these dimensions of SARS-CoV-2 at the time of writing this article; therefore, it was also impossible to estimate the concurrent validity of this questionnaire. Nevertheless, it should be remembered that CoVQuest-CC has been proven and tested on a very large cohort (*n* = 8045) in a general population (from 10 years old to 90 and up). Indeed, it was used in a real-life situation, in the context of the massive screening of the population of the city of Saint-Etienne.

As such, we recommend using the complete version of CoVQuest-CC presented in Table A1 in a descriptive aim to ensure that results will fit with complete and satisfying face and content validity, minus the items Accept_3 (concerning pain assessment for saliva collection with a strong floor effect) and Accept_14 (significantly redundant with Accept_15). to quantify the global knowledges and behaviors about SARS-CoV-2, we recommend using only the questions from the five dimensions presented in Table 3, with respect to the scoring method presented in Table A1 (Appendix A), to calculate the global scores. It is also worth noting that the third factor, “tests screening acceptability”, does not meet the internal consistency quality criteria, with a Cronbach alpha of 0.65 and a ceiling effect, and should therefore mostly be used for descriptive purposes. Finally, scores concerning the factors 2, 4 and 5 must be adapted to certain conditions, such as people not having children, people currently unemployed and people who never had a nasopharyngeal swab.

As West et al. pointed out, adherence to social distancing and isolation behaviors faces strong practical, motivational, and social barriers, while imposing significant costs on the individual and the collective [9]. Moreover, it has been shown that vulnerable, disadvantaged individuals experience these constraints in a more complicated way, particularly in terms of isolation and quarantine [36]. It is by considering these factual elements that we wished to develop and validate the CoVQuest-CC questionnaire. Unfortunately, the pandemic is still ongoing throughout the world, and is being expressed in a virulent manner with the delta variant and recently with the omicron variant [37]. The measures deployed, such as vaccination and screening, are clearly insufficient to stop the spread and contamination of the virus, with the delta and omicron variants being more contagious. Therefore, the adherence of the general population to barrier measures is more necessary than ever. This can only be achieved through an understanding of the stakes involved in getting vaccinated, and adhering to barrier measures and the various public health measures put in place.

Thus, for all these reasons, CoVQuest-CC will play a role in the months to come, to question the level of knowledge and behavioral aspects of individuals in their experience of this health crisis and to deliver them adapted public health messages. This questionnaire can be used to assist the general population in public health actions aiming to work on the commitment and the adhesion of the population towards an individual implementation of protective behaviors with regard to the transmission and propagation of the SARS-Cov2 virus. In the same way, this questionnaire can be a tool of choice for teams working in health promotion and/or health education, in schools with adolescents, or in actions carried out by local authorities with adolescents and young adults. CoVQuest-CC can be used as a screening tool to identify low health literacy levels with respect to COVID-19, and then to structure a public health action aimed at reinforcing this level in order to improve adherence to and enforce a better understanding of health messages and measures to combat the spread and transmission of the virus. In summary, this questionnaire can be used by any professional (e.g., health care provider, researcher, educator) involved in a strategy whose objective is to evaluate and then implement an action aimed at reinforcing the level of health literacy related to COVID-19, from the perspective of a better commitment of the individual to protect him/herself and the collective from the spread and transmission of the virus.

Therefore, the use of CoVQuest-CC should be tested more widely in the French general population through complementary research carried out in other territorial basins, to improve our understanding of the behaviors and knowledge of individuals regarding this type of health emergency. This should also improve our understanding of the behaviors and knowledge of individuals with respect to this type of health emergency. Results on a larger scale on the French population would allow us to establish the levels of knowledge about SARS-CoV-2, as well as levels of adherence to the sanitary measures recommended by the government. Therefore, the analysis of these data would allow us to tailor communication strategies and contents in the framework of public health campaigns to face an epidemic such as SARS-CoV-2.

Finally, we think it is important to remember that the city of Saint-Etienne has been listed several times as having the highest incidence rate in France. The positivity rate was also the highest on several occasions [38]. to deal with the health crisis linked to the pandemic, the health care and scientific communities had to act very quickly and pragmatically. In the end, our experience with the elaboration of this questionnaire, built in a multidisciplinary way, in a very short time, is positive. We were able to demonstrate that it is possible, in a context turned upside down by an unprecedented epidemic for practicing health professionals, to break the academic community out of the silos of the hospital, in responding to an urgent public health problem outside the walls of the hospital. We have managed to bring together all the multidisciplinary workforces needed to co-construct and produce in a thoughtful and rigorous manner a questionnaire. Veresiu and Robinson pointed out that SARS-CoV-2 has totally disrupted, at the global level, the daily life and forecasting capacities of individuals and the collective, as much in terms of health as in terms of the economy, human relations, the educational system, and commerce, among other areas. They underlined that it is not surprising that this global crisis has changed the way people receive and interpret public health messages [39]. Due to this context, the final analysis of the results of the mass screening campaign using the CoVQuest-CC will allow us to supplement the research with additional knowledge on the subject.

## 5. Conclusions

CoVQuest-CC is, according to our knowledge, the first self-administrated French-specific questionnaire investigating the general population’s knowledge and attitudes towards SARS-CoV-2. It has been developed and shown to be understandable by the target population. CoVQuest-CC is valid, consistent, and reliable. Thus, it can be used by Public Health teams or caregivers for public health and research purposes, particularly in the current pandemic context, to evaluate and then implement an action to strengthen the level of health literacy related to COVID-19, with a view to better engaging individuals to protect themselves and the community from the spread and transmission of the virus.

## Figures and Tables

**Table 1:** Overview of the main content of the CoVQuest-CC. Below presents an overview of the main content of the CoVQuest-CC.

**I. Assessment of Your Knowledge of the Modes of Transmission of the Virus** Among these examples from everyday life, please evaluate the risk of transmission of the virus for each of them:
Knowl_1: Talking to 3 people, all masked, for 5 min, in a room without windows
Knowl_2: Talking to 4 people for 30 min, outside, none of them being masked and all of them being 1 m away from each other
Knowl_3: Singing without a mask to 25 people for 30 min in a choir in a large room
Knowl_4: Having a meeting with 10 people, all masked, for 2 h, in a small room (<15 m^2^) without windows
Knowl_5: Having a meal with 8 friends, for 3 h indoors, with a window ajar
Knowl_6: Having an aperitif with 4 friends during an evening, outside on a crowded café terrace
In your opinion, coronavirus can be transmitted:
Knowl_7: Through the air
Knowl_8: Through sputum
Knowl_9: By hands
Knowl_10: Through blood
**II. Assessment of Your Current Behavior with Respect to Barrier Gestures**
Barriere_1: How often do you wear the mask in everyday life in your home when entertaining people who do not live in your home (children, grandchildren, friends, neighbors…)?
Barriere_2: When your are outside, do you SYSTEMATICALLY perform hand hygiene with a hydro-alcoholic solution (after touching money, surfaces when shopping, public transportation seats,…)?
Barriere_3: OTHER THAN using hydro-alcoholic solution, how often have you washed your hands since the beginning of the pandemic?
Barriere_4: How often do you think you respect the physical distance (>1 m), in your life outside (shopping, social life…)?
Barriere_5: How often do you consider respecting the physical distance (at least 1 m) in your indoor life when you receive people who do not live under the same roof (family meals, meals with friends, visits from children, grandchildren…)?
Do you agree with the following statements:
Barriere_6: I limit the number of people I interact with in my PERSONAL life
Barriere_7: I limit the number of people I interact with in my PROFESSIONAL life
**III. Assessment of Your Possible Behavior If You Tested Positive for the Virus**
Isol_1: If you were to isolate yourself for 7–10 days in case of a positive result, what would be your level of concern?
If you were to isolate yourself for 7–10 days in case of a positive result, would you be able to implement the following measures:
Isol_2: Stay for 7 days, for as long as possible (day and night), alone in a room (with no physical contact with those around you)
Isol_3: Get someone to do the shopping
Isol_4: Get someone to make the meal
Isol_5: Get someone to take care of the children
Isol_6: Use a restroom that would be reserved for you
Isol_7: Use a mask at home in the presence of others
Isol_8: Clean several times a day the affected surfaces (door handles, stair railings, light switches,…)
**IV. Tests For Screening**
Accept_1: Regarding the nasopharyngeal screening test (IF YOU HAVE EVER HAD IT), on a scale of 0 to 10, would you say it was?
Accept_2: Regarding nasopharyngeal swabbing (IF YOU HAVE EVER HAD IT), on a scale of 0 to 10, would you say it was?
Accept_3: Regarding the saliva collection, on a scale of 0 to 10, would you say it was?
Accept_4: Regarding saliva collection, on a scale of 0 to 10, would you say it was?
Accept_5: Regarding the saliva and anterior nose swab, on a scale of 0 to 10, would you say it was?
Accept_6: Concerning the self-sampling of saliva and anterior nose, on a scale of 0 to 10, would you say that it was?
Accept_7: If you were to take a new test, which test would you prefer to take?
Accept_8: If you were to be tested again with a test in the front part of your nose in the next few days, what would you do?
Accept_9: If no, why?
Accept_10: If you were to be retested with a saliva test in the next few days, what would you do?
Accept_11: If no, why?
Accept_12: If you were to have a saliva test and a test in the front part of your nose again in the next few days, what would you do?
Accept_13: If no, why?
Accept_14: Would you feel able to take a salivary sputum sample alone in a jar at home and then take it to the laboratory?
Accept_15: Would you feel able to do the self-sampling of salivary sputum and anterior nose alone in a jar at home and then take it to the laboratory?

**Table 2:** Socio-demographic characteristics of respondents.

Characteristics	January (*n* = 3338,%)	February (*n* = 4707, %)	Total (*n* = 8045, %)
Age (years)			
10–19	157 (4.7)	272 (5.8)	429 (5.3)
20–29	359 (10.8)	604 (12.8)	963 (12.0)
30–39	345 (10.4)	524 (11.1)	869 (10.8)
40–49	442 (13.3)	756 (16.1)	1198 (14.9)
50–59	496 (14.9)	810 (17.2)	1306 (16.3)
60–69	698 (21.0)	899 (19.1)	1597 (19.9)
70–79	614 (18.5)	695 (14.8)	1309 (16.3)
80–89	188 (5.7)	136 (2.9)	324 (4.0)
More than 90	24 (0.7)	7 (0.1)	31 (0.4)
Gender			
Female	1704 (51.9)	2634 (56.1)	4338 (54.4)
Male	1581 (48.1)	2058 (43.9)	3639 (45.6)
Profession			
School student	15 (0.5)	152 (3.3)	167 (2.1)
Student	294 (8.9)	370 (7.9)	664 (8.3)
Unemployed	256 (7.8)	234 (5.0)	490 (6.2)
Healthcare Worker	174 (5.3)	464 (9.9)	638 (8.0)
Employee	1025 (31.1)	1584 (33.9)	2609 (32.8)
Self-employed	109 (3.3)	142 (3.0)	251 (3.2)
Retired	1290 (39.2)	1474 (31.5)	2764 (34.7)
Other	129 (3.9)	254 (5.4)	383 (4.8)

**Table 3:** Internal consistency results of the final model.

Subscales	Items	Factor Loading	Cronbach Alpha [95% CI]
SARS-CoV-2 transmission knowledge	Knowl_2	0.458	0.83 [0.81–0.84]
	Knowl_3	0.815	
	Knowl_4	0.732	
	Knowl_5	0.856	
	Knowl_6	0.812	
	Knowl_8	0.212	
Barrier gestures respect	Barriere_1	0.564	0.71 [0.69–0.73]
	Barriere_2	0.364	
	Barriere_4	0.547	
	Barriere_5	0.675	
	Barriere_6	0.58	
	Barriere_7	0.472	
Screening tests acceptability	Accept_8	0.448	0.65 [0.63–0.69]
	Accept_10	0.822	
	Accept_12	0.928	
	Accept_15	0.336	
Home isolation possibility	Isol_2	0.825	0.72 [0.69–0.74]
	Isol_3	0.818	
	Isol_4	0.602	
	Isol_5	0.29	
Tests practicability	Accept_2	0.504	0.83 [0.81–0.84]
	Accept_4	0.848	
	Accept_6	0.876	

## Data Availability

The data presented in this study are available on request from the corresponding author. The data are not publicly available due to: at the time of submission for approval of the research project by the Ethics Committee, there was no stipulation that the data would be open access.

## Appendix A

**Table A1:** Content of the CoVQuest-CC questionnaire after initial development.

**I. Evaluation De Vos Connaisssances Sur Les Modes De Transmission Du Virus** **Free translation for English-speaking readers: Assessment of your knowledge of the modes of transmission of the virus** Parmi ces exemples de la vie courante, merci d’évaluer le risque de transmission du virus pour chacune d’entre elles (“Nul” = sans risque de transmission a “Très important” = risque très fort de transmission): ***Free translation for English-speaking readers: Among these examples from everyday life, please evaluate the risk of transmission of the virus for each of them (“Nil” = no risk of transmission a “Very important” = very high risk of transmission):*** **Knowl_1**: Discuter a 3 personnes, toutes masquées, pendant 5 min, dans une pièce sans fenêtre] ***Free translation for English-speaking readers: Talking to 3 people, all masked, for 5 min, in a room without windows*** -Nul (0.5) -Très faible (1) -Faible (1) -Modéré (0.5) -Important (0) -Très important (0)
**Knowl_2**: Discuter a 4 personnes pendant 30 min, à l’extérieur, aucune n’étant masquée et toutes sont à 1 m les unes des autres. ***Free translation for English-speaking readers: Talking to 4 people for 30 min, outside, none of them being masked and all of them being 1 m away from each other*** -Nul (0) -Très faible (0) -Faible (0.5) -Modéré (1) -Important (1) -Très important (0.5)
**Knowl_3**: Chanter sans masque a 25 personnes pendant 30 min en chorale dans une grande salle. ***Free translation for English-speaking readers: Singing without a mask to 25 people for 30 min in a choir in a large room*** -Nul (0) -Très faible (0) -Faible (0) -Modéré (0.5) -Important (1) -Très important (1)
**Knowl_4**: Faire une réunion a 10 personnes, toutes masquées, pendant 2 h, dans une petite pièce (<15 m^2^) sans fenêtre. ***Free translation for English-speaking readers: Having a meeting with 10 people, all masked, for 2 h, in a small room (<15 m^2^) without windows*** -Nul (0) -Très faible (0) -Faible (0) -Modéré (0.5) -Important (1) -Très important (1)
**Knowl_5**: Prendre un repas avec 8 amis, pour une durée de 3 heures à l’intérieur, avec une fenêtre entre-ouverte. ***Free translation for English-speaking readers: Having a meal with 8 friends, for 3 h indoors, with a window ajar*** -Nul (0) -Très faible (0) -Faible (0) -Modéré (0.5) -Important (1) -Très important (1)
**Knowl_6**: Prendre un apéritif avec 4 amis au cours d’une soirée, à l’extérieur sur une terrasse de café bondée. ***Free translation for English-speaking readers: Having an aperitif with 4 friends during an evening, outside on a crowded café terrace*** -Nul (0) -Très faible (0) -Faible (0) -Modéré (0.5) -Important (1) -Très important (1)
**Knowl_7**: Selon vous, le coronavirus peut se transmettre: [Par l’air] ***Free translation for English-speaking readers: In your opinion, coronavirus can be transmitted: [Through the air]*** -Non (0) -Oui (1) -Ne sais pas (0)
**Knowl_8**: Selon vous, le coronavirus peut se transmettre: [Par les postillons] ***Free translation for English-speaking readers: In your opinion, coronavirus can be transmitted: [Through sputum]*** -Non (0) -Oui (1) -Ne sais pas (0)
**Knowl_9**: Selon vous, le coronavirus peut se transmettre: [Par les mains] ***Free translation for English-speaking readers: In your opinion, coronavirus can be transmitted: [By hands]*** -Non (0) -Oui (1) -Ne sais pas (0)
**Knowl_10**: Selon vous, le coronavirus peut se transmettre: [Par le sang] ***Free translation for English-speaking readers: In your opinion, coronavirus can be transmitted: [Through blood]*** -Non (0) -Oui (1) -Ne sais pas (0)
**II. EVALUATION DE VOTRE COMPORTEMENT VIS A VIS DES GESTES BARRIERES** ***Free translation for English-speaking readers: Assessment of your current behavior with respect to barrier gestures*** **Barriere_1**: A quelle fréquence portez-vous le masque dans la vie de tous les jours à votre domicile quand vous recevez des gens qui ne vivent pas sous votre toit (enfants, petits-enfants, amis, voisins…) ? ***Free translation for English-speaking readers: How often do you wear the mask in everyday life in your home when entertaining people who do not live in your home (children, grandchildren, friends, neighbors…)?*** -Jamais (0) -De temps en temps (1) -La moitié du temps (2) -Souvent (3) -Tout le temps (4)
**Barriere_2**: A l’extérieur, faites-vous une hygiène des mains avec une solution hydro-alcoolique SYSTEMATIQUEMENT (après avoir touché de l’argent, surfaces lors de courses, sièges des transports en commun, …)? ***Free translation for English-speaking readers: When your are outside, do you SYSTEMATICALLY perform hand hygiene with a hydro-alcoholic solution (after touching money, surfaces when shopping, public transportation seats, …)?*** -Jamais (0) -De temps en temps (1) -La moitié du temps (2) -Souvent (3) -Tout le temps (4)
**Barriere_3**: EN DEHORS de l’utilisation de solution hydro-alcoolique, à quelle fréquence vous lavez-vous les mains depuis le début de la pandémie ? ***Free translation for English-speaking readers: OTHER THAN using hydro-alcoholic solution, how often have you washed your hands since the beginning of the pandemic?*** -Jamais (0) -De temps en temps (1) -La moitié du temps (2) -Souvent (3) -Tout le temps (4)
**Barriere_4**: A quelle fréquence pensez-vous respecter la distanciation physique (>1 m), dans votre vie a l’exterieur (courses, vie sociale…) ? ***Free translation for English-speaking readers: How often do you think you respect the physical distance (>1 m), in your life outside (shopping, social life...)?*** -Jamais (0) -De temps en temps (1) -La moitié du temps (2) -Souvent (3) -Tout le temps (4)
**Barriere_5**: A quelle fréquence considérez-vous respecter la distanciation physique (au moins 1 m) dans votre vie a l’intérieur lorsque vous recevez des personnes qui n’habitent pas sous le même toit (repas de famille, repas avec des amis, visite des enfants, petits-enfants…) ? ***Free translation for English-speaking readers: How often do you consider respecting the physical distance (at least 1 m) in your indoor life when you receive people who do not live under the same roof (family meals, meals with friends, visits from children, grandchildren…)?*** -Jamais (0) -De temps en temps (1) -La moitié du temps (2) -Souvent (3) -Tout le temps (4)
**Barriere_6**: Etes-vous d’accord avec les affirmations suivantes: [“Je limite le nombre de personnes avec lesquelles j’interagis dans ma vie PERSONNELLE”] ***Free translation for English-speaking readers: Do you agree with the following statements: [“I limit the number of people I interact with in my PERSONAL life”]*** -Pas du tout d’accord (0) -Pas d’accord (1) -Ni plus, ni moins d’accord (2) -D’accord (3) -Tout a fait d’accord (4)
**Barriere_7**: Etes-vous d’accord avec les affirmations suivantes: [“Je limite le nombre de personnes avec lesquelles j’interagis dans ma vie PROFESSIONNELLE”] ***Free translation for English-speaking readers: Do you agree with the following statements: [“I limit the number of people I interact with in my PROFESSIONAL life”]*** -Pas du tout d’accord (0) -Pas d’accord (1) -Ni plus, ni moins d’accord (2) -D’accord (3) -Tout à fait d’accord (4)
**III. EVALUATION DE VOTRE COMPORTEMENT POSSIBLE SI VOUS ETIEZ DEPISTE POSITIF** ***Free translation for English-speaking readers: Assessment of your possible behavior if you tested positive for the virus*** **Isol_1**: Si vous deviez vous isoler pendant 7 à 10 jours en cas de résultat positif, quel serait votre niveau d’inquiétude: ***Free translation for English-speaking readers: If you were to isolate yourself for 7–10 days in case of a positive result, what would be your level of concern:*** -Angoisse(e) (4) -Très inquiet(e) (3) -Inquiet(e) (2) -Peu inquiet(e) (1) -Pas inquiet(e) (0)
**Isol_2**: Si vous deviez vous isoler pendant 7 à 10 jours en cas de résultat positif, vous serait-il possible de mettre en œuvre les mesures suivantes: [Rester pendant 7 jours, le plus longtemps possible (jour et nuit), seul(e) dans une pièce (sans avoir de contacts physiques avec votre entourage)] ***Free translation for English-speaking readers: If you were to isolate yourself for 7–10 days in case of a positive result, would you be able to implement the following measures: [Stay for 7 days, for as long as possible (day and night), alone in a room (with no physical contact with those around you)]*** -Impossible (4) -Très difficile (3) -Difficile (2) -Facile (1) -Très facile (0)
**Isol_3**: Si vous deviez vous isoler pendant 7 à 10 jours en cas de résultat positif, vous serait-il possible de mettre en œuvre les mesures suivantes: [Faire intervenir quelqu’un pour faire les courses] ***Free translation for English-speaking readers: If you were to isolate yourself for 7–10 days if you tested positive, would you be able to implement the following measures: [Get someone to do the shopping]*** -Impossible (4) -Très difficile (3) -Difficile (2) -Facile (1) -Très facile (0)
**Isol_4**: Si vous deviez vous isoler pendant 7 à 10 jours en cas de résultat positif, vous serait-il possible de mettre en œuvre les mesures suivantes: [Faire intervenir quelqu’un pour faire le repas] ***Free translation for English-speaking readers: If you were to isolate yourself for 7–10 days if you tested positive, would you be able to implement the following measures: [Get someone to make the meal]*** -Impossible (4) -Très difficile (3) -Difficile (2) -Facile (1) -Très facile (0)
**Isol_5**: Si vous deviez vous isoler pendant 7 à 10 jours en cas de résultat positif, vous serait-il possible de mettre en œuvre les mesures suivantes: [Faire intervenir quelqu’un pour prendre en charge les enfants] ***Free translation for English-speaking readers: If you were to isolate yourself for 7–10 days in the event of a positive result, would you be able to implement the following measures: [Get someone to take care of the children]*** -Impossible (4) -Très difficile (3) -Difficile (2) -Facile (1) -Très facile (0)
**Isol_6**: Si vous deviez vous isoler pendant 7 à 10 jours en cas de résultat positif, vous serait-il possible de mettre en œuvre les mesures suivantes: [Utilisez des toilettes qui vous seraient réservées] ***Free translation for English-speaking readers: If you were to isolate yourself for 7–10 days in the event of a positive result, would you be able to implement the following measures: [Use a restroom that would be reserved for you]*** -Impossible (4) -Très difficile (3) -Difficile (2) -Facile (1) -Très facile (0)
**Isol_7**: Si vous deviez vous isoler pendant 7 à 10 jours en cas de résultat positif, vous serait-il possible de mettre en œuvre les mesures suivantes: [Utilisez un masque à domicile en présence de votre entourage] ***Free translation for English-speaking readers: If you were to isolate yourself for 7–10 days in the event of a positive result, would you be able to implement the following measures: [Use a mask at home in the presence of others]*** -Impossible (4) -Très difficile (3) -Difficile (2) -Facile (1) -Très facile (0)
**Isol_8**: Si vous deviez vous isoler pendant 7 a 10 jours en cas de résultat positif, vous serait-il possible de mettre en œuvre les mesures suivantes: [Nettoyer les surfaces plusieurs fois par jour les surfaces touchées (poignées de porte, rampes d’escalier, interrupteurs, …)] ***Free translation for English-speaking readers: If you were to isolate yourself for 7 to 10 days in case of a positive result, would you be able to implement the following measures: [Clean the surfaces several times a day the affected surfaces (door handles, stair railings, light switches, …)]*** -Impossible (4) -Très difficile (3) -Difficile (2) -Facile (1) -Très facile (0)
**IV. LES PRELEVEMENTS** ***Free translation for English-speaking readers: Tests for screening*** **Accept_1**: Concernant le prélèvement nasopharyngé (SI VOUS EN AVEZ DEJA BENEFICIE), sur une échelle de 0 a 10, diriez-vous qu’il était: [Douloureux (0 = pas douloureux du tout à 10 = pire douleur imaginable)] ***Free translation for English-speaking readers: Regarding the nasopharyngeal swab (IF YOU HAVE EVER HAD IT), on a scale of 0 to 10, would you say it was: [Painful (0 = not painful at all to10 = worst pain imaginable)]***
**Accept_2**: Concernant le prélèvement nasopharyngé (SI VOUS EN AVEZ DEJA BENEFICIE), sur une échelle de 0 a 10, diriez-vous qu’il était: [Pratique (facile, rapide — 0 = pas pratique du tout à 10 = extrêmement pratique)] ***Free translation for English-speaking readers: Regarding nasopharyngeal swabbing (IF YOU HAVE EVER HAD IT), on a scale of 0 to 10, would you say it was: [Convenient (easy, quick - 0 = not convenient at all to 10 = extremely convenient)]***
**Accept_3**: Concernant le prélèvement salivaire, sur une échelle de 0 a 10, diriez-vous qu’il était: [Douloureux (0 = pas douloureux du tout à 10 = pire douleur imaginable)] ***Free translation for English-speaking readers: Regarding the saliva collection, on a scale of 0 to 10, would you say it was: [Painful (0 = not painful at all to 10 = worst pain imaginable)]***
**Accept_4**: Concernant le prélèvement salivaire, sur une échelle de 0 a 10, diriez-vous qu’il était: [Pratique (facile, rapide - 0 = pas pratique du tout à 10 = extrêmement pratique)] ***Free translation for English-speaking readers: Regarding saliva collection, on a scale of 0 to 10, would you say it was: [Convenient (easy, quick - 0 = not convenient at all to 10 = extremely convenient)]***
**Accept_5**: Concernant le prélèvement l’auto-prélèvement salive et nez antérieur, sur une échelle de 0 a 10, diriez-vous qu’il était: [Douloureux (0 = pas douloureux du tout à 10 = pire douleur imaginable)] ***Free translation for English-speaking readers: Regarding the saliva and anterior nose swab, on a scale of 0 to 10, would you say it was: [Painful (0 = not painful at all to 10 = worst pain imaginable)]***
**Accept_6**: Concernant le prélèvement l’auto-prélèvement salive et nez antérieur, sur une échelle de 0 a 10, diriez-vous qu’il était: [Pratique (facile, rapide — 0 = pas pratique du tout à 10 = extrêmement pratique)] ***Free translation for English-speaking readers: Concerning the self-sampling of saliva and anterior nose, on a scale of 0 to 10, would you say that it was: [Convenient (easy, quick — 0 = not convenient at all to 10 = extremely convenient)]***
**Accept_7**: Si vous deviez faire un nouveau test, quel test préfériez-vous faire? -Pas de préférence (0) -Test sur crachat salivaire (1) -Test sur auto-prélèvement salivaire et nez antérieur (2) -Test classique (dans le nez) (3) ***Free translation for English-speaking readers: If you were to take a new test, which test would you prefer to take?*** ***-No preference (0)*** ***-Saliva sputum test (1)*** ***-Saliva swab and anterior nose test (2)*** ***-Classic test (in the nose) (3)***
**Accept_8**: Si l’on devait vous refaire un test classique (dans le nez) dans les jours à venir, que feriez-vous? -Je refuserais certainement (0) -Je refuserais probablement (1) -J’accepterais probablement (2) -J’accepterais certainement (3) ***Free translation for English-speaking readers: If you were to be tested again with a test in the front part of your nose in the next few days, what would you do?*** ***-I would definitely refuse (0)*** ***-I would probably refuse (1)*** ***-I would probably accept (2)*** ***-I would definitely accept (3)***
**Accept_9**: Si refus, pourquoi? -Autre -Difficultés pratiques pour effectuer l’examen (délais de rendez-vous, temps d’attente, etc.) -Examen trop désagréable, douloureux ***Free translation for English-speaking readers: If no, why?*** ***-Other*** ***-Practical difficulties to perform the examination (appointment delays, waiting time, etc.)*** ***-Examination too unpleasant, painful***
**Accept_10**: Si l’on devait vous refaire un test salivaire dans les jours a venir, que feriez-vous? -Je refuserais certainement (0) -Je refuserais probablement (1) -J’accepterais probablement (2) -J’accepterais certainement (3) ***Free translation for English-speaking readers: If you were to be retested with a saliva test in the next few days, what would you do?*** ***-I would definitely refuse (0)*** ***-I would probably refuse (1)*** ***-I would probably accept (2)*** ***-I would definitely accept (3)***
**Accept_11**: Si refus, pourquoi? -Autre -Difficultés pratiques pour effectuer l’examen (délais de rendez-vous, temps d’attente, etc.) -Examen trop désagréable, douloureux ***Free translation for English-speaking readers: If no, why?*** ***-Other*** ***-Practical difficulties to perform the examination (appointment delays, waiting time, etc.)*** ***-Examination too unpleasant, painful***
**Accept_12**: Si l’on devait vous refaire un test salivaire et nez anterieur dans les jours a venir, que feriez-vous? -Je refuserais certainement (0) -Je refuserais probablement (1) -J’accepterais probablement (2) -J’accepterais certainement (3) ***Free translation for English-speaking readers: If you were to have a saliva test and a test in the front part of your nose again in the next few days, what would you do?*** ***-I would definitely refuse (0)*** ***-I would probably refuse (1)*** ***-I would probably accept (2)*** ***-I would definitely accept (3)***
**Accept_13**: Si refus, pourquoi? -Autre -Difficultés pratiques pour effectuer l’examen (délais de rendez-vous, temps d’attente, etc.) -Examen trop désagréable, douloureux ***Free translation for English-speaking readers: If no, why?*** ***-Other*** ***-Practical difficulties to perform the examination (appointment delays, waiting time, etc.)*** ***-Examination too unpleasant, painful***
**Accept_14**: Vous sentiriez-vous capable de faire le prélèvement de crachat salivaire seul dans un pot chez vous puis le déposer au laboratoire? -Certainement non (0) -Probablement non (1) -Probablement oui (2) -Certainement oui (3) ***Free translation for English-speaking readers: Would you feel able to take a salivary sputum sample alone in a jar at home and then take it to the laboratory?*** ***-Certainly not (0)*** ***-Probably no (1)*** ***-Probably yes (2)*** ***-Definitely yes (3)***
**Accept_15**: Vous sentiriez-vous capable de faire l’auto prélèvement de crachat salivaire et nez antérieur seul dans un pot chez vous puis le déposer au laboratoire? -Certainement non (0) -Probablement non (1) -Probablement oui (2) -Certainement oui (3) ***Free translation for English-speaking readers: Would you feel able to do the self-sampling of salivary sputum and anterior nose alone in a jar at home and then take it to the laboratory?*** ***-Certainly not (0)*** ***-Probably no (1)*** ***-Probably yes (2)*** ***-Certainly yes (3)***
-Questions acronyms are indicated in bold type. The only purpose of these acronyms is to refer more easily to the questions in this article, and they therefore were not present in distributed questionnaires. -All categorical questions integrated in the internal consistency validation procedure were converted into numerical variables for the purpose of this procedure. For these variables, numerical conversion values are indicated in brackets next to each modality. These values were not indincated in the distributed questionnaires. -People responding “Autre” at Accept_9, Accept_11 and Accept_13 were given the possibility to precise their reason in a free-form text
